# A Combination of Alkaloids and Triterpenes of *Alstonia scholaris* (Linn.) R. Br. Leaves Enhances Immunomodulatory Activity in C57BL/6 Mice and Induces Apoptosis in the A549 Cell Line

**DOI:** 10.3390/molecules181113920

**Published:** 2013-11-12

**Authors:** Liang Feng, Yan Chen, Ling Yuan, Xuan Liu, Jun-Fei Gu, Ming-Hua Zhang, Ying Wang

**Affiliations:** Key Laboratory of New Drug Delivery Systems of Chinese Meteria Medica, Jiangsu Provincial Academy of Chinese Medicine, Nanjing 210028, Jiangsu, China; E-Mails: wenmoxiushi@163.com (L.F.); yuanl.china@sina.com (L.Y.); liu_xuan1987@163.com (X.L.); gujunfei0123@126.com (J.-F.G.); jdyx0701.111@163.com (M.-H.Z.); wangying9021@163.com (Y.W.)

**Keywords:** *Alstonia scholaris* (Linn.) R. Br., antitumor, apoptosis, immunomodulatory, non-small cell lung cancer

## Abstract

Experiments were conducted to evaluate the induction of apoptosis and the immunomodulatory activities of alkaloids and triterpenes of *Alstonia scholaris* (Linn.) R. Br. leaves (ASL). Importantly, their possible synergistic properties were also explored in this study. Human lung adenocarcinoma cell line A549 and Lewis tumor-bearing C57BL/6 mice were used for the evaluation of their activities. A MTT assay was used to determine the proliferation inhibition in A549 cells. Annexin-V/PI double staining as well as flow cytometry was performed to detect apoptosis and cell cycle status. Enzyme-linked immunosorbent assay (ELISA) was conducted to determine the levels of inflammatory mediators interleukin-6 (IL-6) and tumor necrosis factor-α (TNF-α) in serum. Furthermore, western blot analysis was applied to evaluate the expressions of proteins associated with cell death. Alkaloids or triterpenes showed a high anti-proliferative activity in A549 cells, with IC_50_ values of 14.4 µg/mL and 9.3 µg/mL, respectively. The alkaloids and triterpenes combination could significantly inhibit tumor growth in tumor-bearing C57BL/6 mice, compared with alkaloids or triterpenes alone (7.5, 15, 30 g raw material/kg). The immune organs indexes including spleen index and thymus index were increased remarkably by the combination of alkaloids and triterpenes, whereas the levels of IL-6 and TNF-α were up-regulated significantly. Moreover, Annexin-V/PI double staining and flow cytometry showed that the combination of alkaloids and triterpenes (1, 2 and 3 mg raw material/kg) could induce apoptosis and cause S cell cycle arrest in A549 cells. Western blot analysis also showed that their combination (2 mg raw material/kg) significantly down-regulated Bcl-2 expression and pro-casp8 level, whereas it remarkably increased the level of cleaved caspase-8 leading to apoptosis in A549 cells. These observations provide preliminary evidence that both alkaloids and triterpenes possess immune regulation and induction apoptosis activities. Their combination has a stronger activity than that of either class alone. Our findings suggested that these components might be beneficial for the prevention and treatment of NSCLC through a significant synergy effect.

## 1. Introduction

Non-small cell lung cancer (NSCLC), one of the most commonly diagnosed malignancies, has been shown to be the leading cause of cancer-related mortality throughout the World [1,2]. Traditional Chinese Medicine (TCM) therapy has been regarded as a beneficial complementary alternative medicine for survival, preventing relapse/metastasis and improving quality of life of NSCLC patients suffering from chemo/radiotherapy-induced side effects [3]. Of note that the synergistic properties of TCM contribute to the prevention and treatment of cancer or tumors, such as NSCLC [4].

TCM is a holistic medicine with emphasis on the integrity of the human body due to its multi-component features [5]. In a Chinese materia medica or a formula, there are multiple components which are responsible for the prevention and treatment of diseases through multiple targets [6]. Based on their chemical structure characteristics, these ingredients can be divided to different classes, such as alkaloids, triterpenoids, polysaccharides, and so on. Many studies have confirmed that these components are beneficial for preventing the onset and progression of the disease by working together [7,8]. The combination of the use of different components may be effective for the prevention and treatment of NSCLC. Accumulating evidence shows that the combination of different components exerted a synergistic stronger anti-tumor activity via multiple mechanisms [9,10].

*Alstonia scholaris* (Linn.) R. Br. (ASL) is a common plant in Yunnan Province of P.R. China. Its leaves have been traditionally used in ‘‘dai” ethnopharmacy to treat chronic respiratory diseases [11]. ASL has been observed to possess chemopreventive, antioxidant, antimutagenic and immunomodulatory activities, all of which are properties efficacious for cancer or tumor treatment [12]. The experimental evidence given directly indicated that ASL had a significant anti-lung cancer activity *in vitro* [13]. Phytochemical researchers have reported that the leaves of ASL contain alkaloids (picrinine, schloaricine, alstonine and rhazimanine, *etc.*) and triterpenes (botulin, ursolic acid, *etc.*) which are responsible for its anti-tumor properties [14,15,16,17]. However, the potential immunomodulatory and apoptosis induction activities of these alkaloids and triterpenes and their combination on NSCLC were not fully understood.

Therefore, the present study was undertaken to evaluate the *in vitro* and *in vivo* immunomodulatory and apoptosis induction activities of the alkaloids and triterpenes of ASL leaves, as well as their combination, and explore the possible synergistic effects of the alkaloids and triterpenes and their preliminary mechanism of action.

## 2. Results and Discussion

### 2.1. Characterization of the Chemical Compounds by HPLC and LC/MS/MS

**Figure 1:**
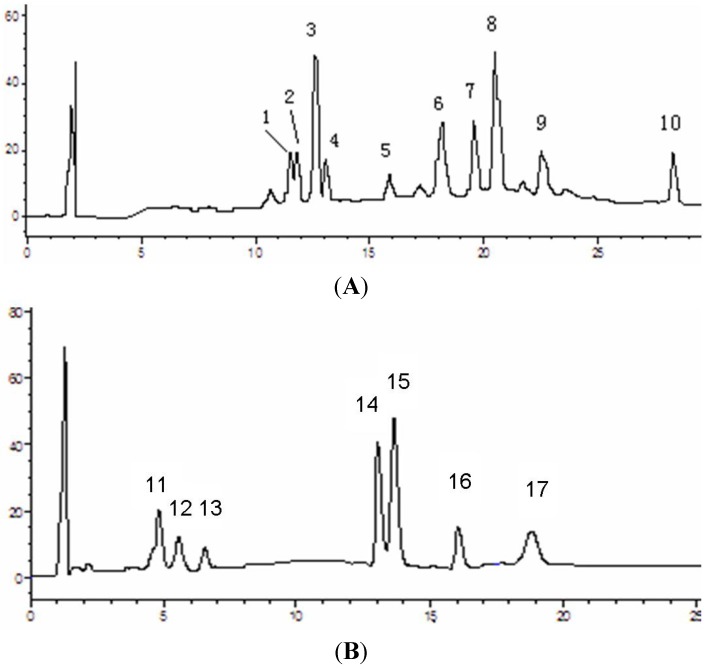
HPLC chromatograms of alkaloids (**A**) and triterpenes (**B**). The detection wavelength for alkaloids were 287 nm while for triterpenes were 220 nm. 1. Scholaricine; 2. 19-Epi-scholaricine; 3. Sarpagine; 4. N4-Demthylechitamine; 5. Echitamidine; 6. Strictamine; 7. Akuammidine; 8. Vallesamine; 9. Picraline; 10. Picralinal; 11. Cylicodiscic acid; 12. Betulin; 13. Betulinic acid; 14. Oleanolic acid; 15. Ursolic acid; 16. Cycloeucalenol; 17. α-amyrin acetate.

ASL, distributed widely in tr opical and subtropical countries, is also used as an ethnomedicine in Yunnan Province of China. The bark and root of this plant have been used as folk medicine for the treatment of cancer in China [18]. In this present study, the leaf of ASL was investigated. The main alkaloids and triterpenes in ASL leaves were identified by HPLC and LC/MS/MS. Ten peaks were shown to be alkaloids, while seven peaks were triterpenes according to HPLC (Figure 1A,B).

**Figure 2:**
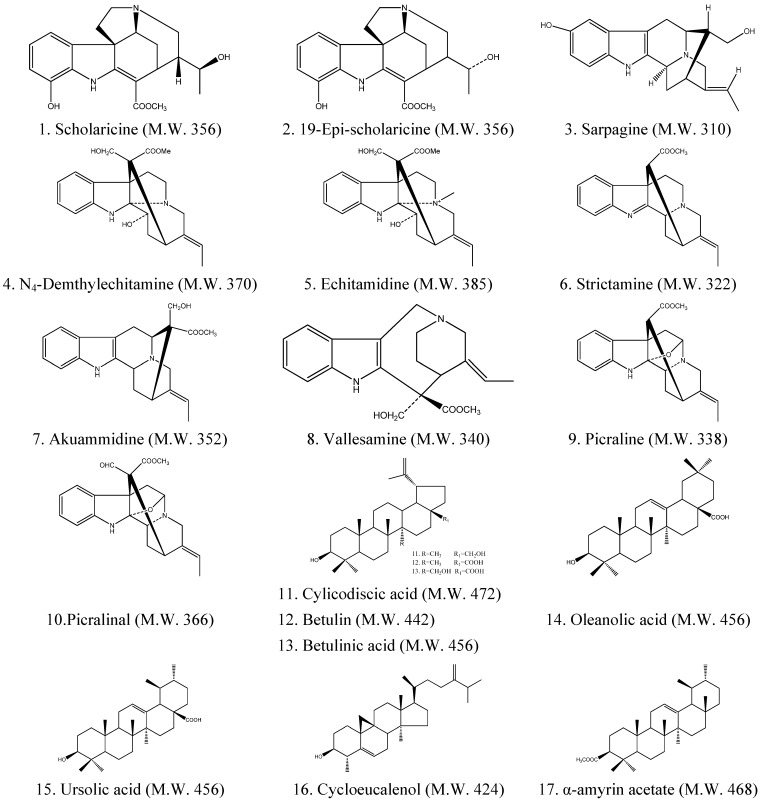
The chemical structures of the identified alkaloid and triterpene compounds.

The (+)ESI-MS *m/z* of alkaloids were 357, 357, 311, 371, 386, 323, 353, 341, 339, 367 while triterpenes were 473, 457, 443, 457, 457, 423, 468. According to the (+)ESI-MS *m/z*, UV_max_ (nm) and ion fragments of these compounds, the 10 alkaloid peaks were identified as scholaricine (5.3%), 19-epi-scholaricine (5.2%), sarpagine (14.6%), N_4_-demthylechitamine (4.1%), echitamidine (2.2%), strictamine (10.7%), akuammidine (9.3%), vallesamine (12.4%), picraline (6.9%) and picralinal (7.2%), respectively, while the seven triterpene peaks were identified as cylicodiscic acid (7.7%), betulin (5.8%), betulinic acid (5.4%), oleanolic acid(15.1%), ursolic acid (23.6%), cycloeucalenol (10.3%) and α-amyrin acetate (6.5%) (Table 1 and Figure 2). These known compounds were the main active ingredients in the leaves of ALS.

**Table 1:** Peak assignment for the components from alkaloids and triterpenes.

No	Classification	Compound identity	T_R_ (min)	(+)ESI-MS *m/z*	UVmax (nm)	Ion fragments	Formula
**1**	Alkaloids	Scholaricine	11.65	357	336, 298, 285, 266	339, 325, 311, 198	C_20_H_24_N_2_O_4_
**2**		19-Epi-scholaricine	11.84	357	360, 311, 293, 286	339, 325, 311, 198	C_20_H_24_N_2_O_4_
**3**		Sarpagine	12.57	311	364, 256	294, 278, 253, 226, 186	C_19_H_22_N_2_O_2_
**4**		N_4_-Demthylechitamine	12.85	371	297, 266	353, 339, 256, 189	C_21_H_26_N_2_O_4_
**5**		Echitamidine	15.83	386	329, 304, 294, 262	3369, 355, 271, 211	C_22_H_29_N_2_O_4_
**6**		Strictamine	18.30	323	365, 261, 239	295, 263, 216, 156	C_20_H_22_N_2_O_2_
**7**		Akuammidine	19.64	353	279, 251	307, 293, 230, 202	C_21_H_24_N_2_O_3_
**8**		Vallesamine	20.63	341	362, 258, 240	323, 309, 267, 108	C_20_H_24_N_2_O_3_
**9**		Picrinine	22.49	339	336, 257, 239	307, 265, 107	C_20_H_22_N_2_O_3_
**10**		Picralinal	28.31	367	365, 259, 240	337, 324, 280, 260	C_21_H_22_N_2_O_4_
**11**	Triterpenoids	Cylicodiscic acid	4.77	473	223, 203	455, 441, 423, 232	C_30_H_48_O_4_
**12**		Betulinic acid	5.54	457	210	441, 429, 411, 220	C_30_H_48_O_3_
**13**		Betulin	6.62	443	208	425, 413, 395, 204	C_30_H_50_O_2_
**14**		Oleanolic acid	13.07	457	205	439, 427, 409, 381, 259, 217	C_30_H_48_O_3_
**15**		Ursolic acid	13.86	457	205	439, 427, 409, 381, 259, 217	C_30_H_48_O_3_
**16**		Cycloeucalenol	16.17	423	200	408, 385, 367,315, 289, 213	C_30_H_48_O
**17**		α-amyrin acetate	18.94	468	225	435, 419, 401, 381	C_32_H_52_O_2_

### 2.2. The Inhibition of Alkaloids, Triterpenes and Their Combination on Cell Proliferation in A549 Cells

**Figure 3:**
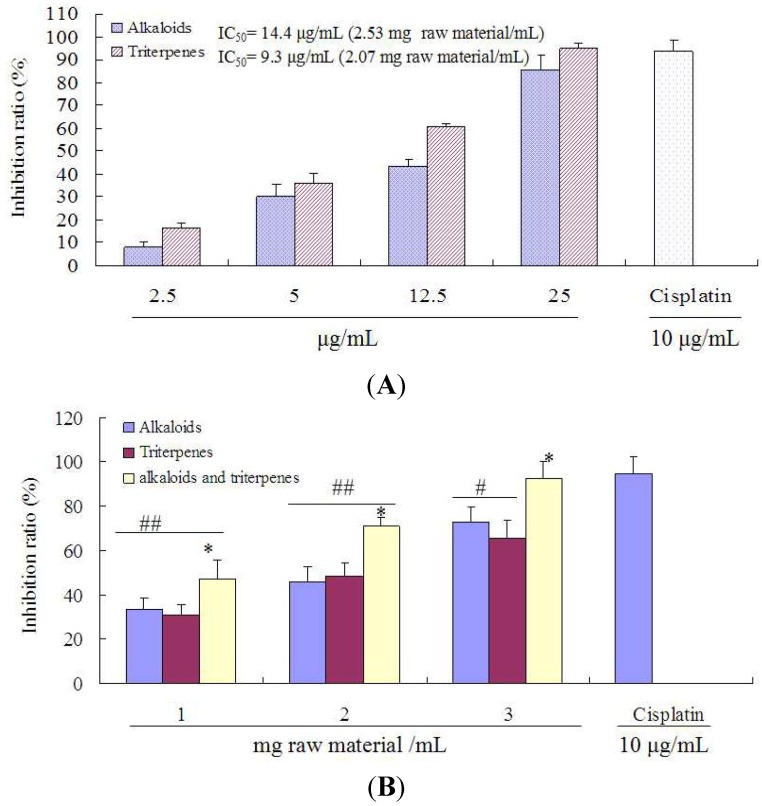
Proliferation inhibition of alkaloids, triterpenes and their combination on the growth in A549 cells. Cells were treated with 2.5, 5, 12.5 and 25 μg/mL alkaloids or triterpenes extract to determine the IC_50_ values (**A**). The IC_50_ is defined as the concentration that results in 50% cell death compared to that of the blank cultures. Additionally, cells were exposed to 1, 2 and 3 mg raw material/mL (**B**) (for alkaloids, 7, 14 and 21 μg/mL extract; for triterpenes, 4.5, 9 and 13.5 μg/mL extract). Cisplatin (10 μg/mL) was chosen as a positive control. The OD values were read at 550 nm. The values represent the means of three independent experiments having similar patterns. * *p* < 0.05, *vs.* alkaloids or triterpenes alone; *^#^* *p* < 0.05, *^##^* *p* < 0.01, *vs.* Cisplatin.

The ethanol extract of the stem bark of ASL has anticancer activity in human cancer cell lines such as HeLa, MCF-7, and HS1 human sarcoma [19,20]. Previous studies have showed that the alkaloid compounds such as picrinine and triterpene compounds such as ursolic acid of ASL have anti-tumor properties in various cancer cell lines [14], therefore, the proliferation inhibition in A549 cells of alkaloids or triterpenes extracted from ASL leaves was studied by an MTT assay. In order to compare the differences between the components alone and their combination, the doses of alkaloids, triterpenes or their combination were calculated as raw material/kg. As shown in Figure 3A, the treatment with alkaloids or triterpenes (2.5, 5, 12.5 and 25 μg/mL extract) significantly inhibited the proliferation of A549 cells in a concentration-dependent manner. 

Notably, the IC_50_ values of alkaloids and triterpenes against A549 cells were 14.4 μg/mL (2.53 mg raw material/mL) and 9.3 μg/mL (2.07 mg raw material/mL), respectively. These results showed that alkaloids and triterpenes of ASL leaves had strong proliferation inhibition activity in human lung cancer A549 cells. In order to evaluate whether alkaloids and triterpenes can inhibit A549 cell proliferation through their synergistic effects, the activity differences between alkaloids or triterpenes alone and their combination were compared. As depicted in Figure 3B, a significant increase was observed after treatment with their combination (1, 2, 3 mg raw material/mL), compared to alkaloids or triterpenes alone, at equal doses of raw material (*p* < 0.05). These results suggest that the combined administration of alkaloids and triterpenes may enhance the inhibition on A549 cells proliferation through their synergistic effects.

For single components, the concentration of component can be calculated in μg/mL, however, for the combination of two components, the dose can be calculated as the amount of raw material. Particularly, when the combination of the two components is considered, if the dose unit is μg/mL rather than mg raw material/mL, the difference between a single dose and the combination cannot be compared due to their inconsistency. Therefore, in this study, the single component units in Figure 3A were changed to the μg/mL and mg raw material/mL.

### 2.3. The Inhibition of Alkaloids, Triterpenes and Their Combination on Tumor Growth in C57BL/6 Mice

**Figure 4:**
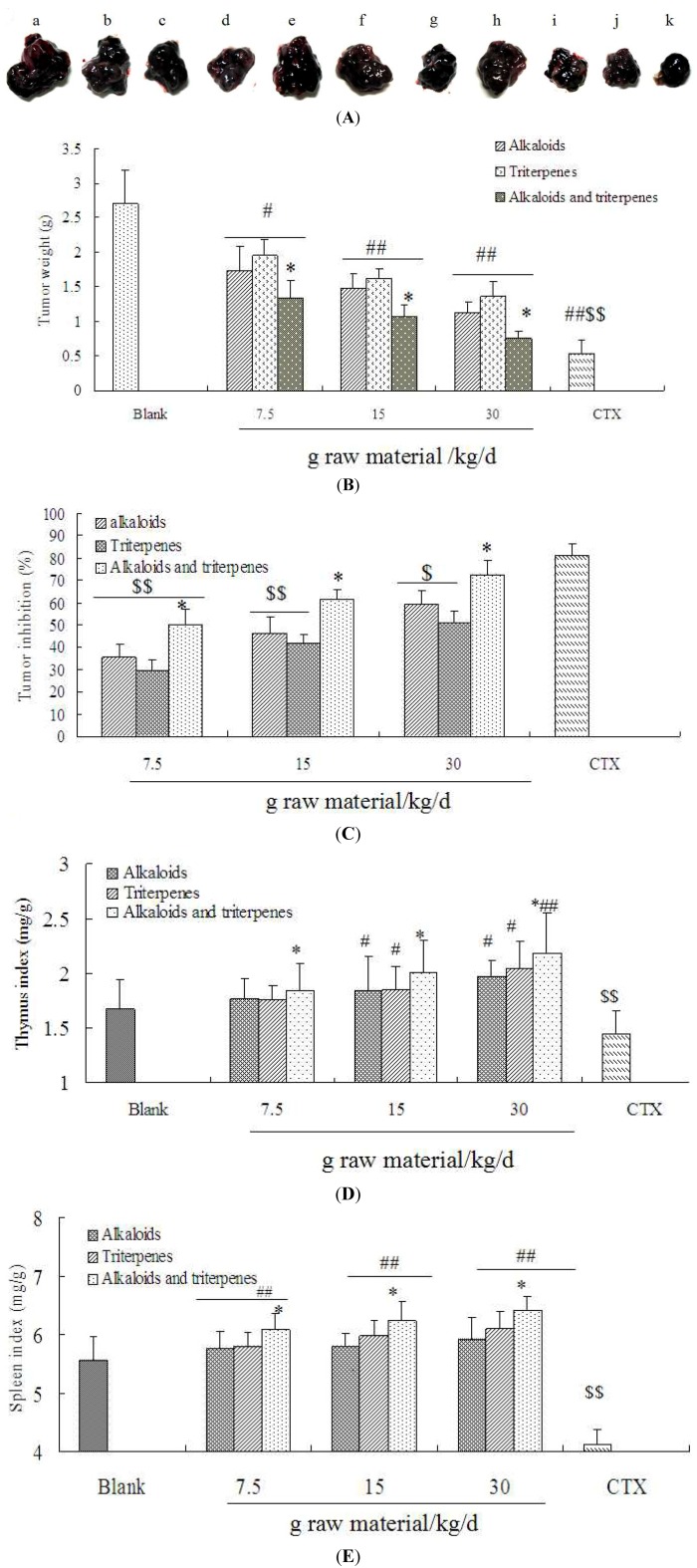
Tumor inhibition and immune organic indexes of alkaloids, triterpenes and their combination on Lewis lung carcinoma cell-bearing C57BL/6 mice. Mice were subcutaneously injected 0.2 mL Lewis cells and then administered orally with alkaloids, triterpenes or their combination (7.5, 15, 30 g raw material/kg/d) for continuous 14 days. Tumors excised from C57BL/6 mice are exhibited in (**A**): blank control (**a**); 7.5, 15, 30 g raw material/kg/d alkaloids (**b**–**d**); 7.5, 15, 30 g raw material/kg/d triterpenes (**e**–**g**); 7.5, 15, 30 g raw material/kg/d their combination (**h**–**j**); CTX of 20 mg/kg/d was administered by intraperitoneal injection (**k**). Tumor weight and inhibition rates are shown in (**B**) and (**C**) while thymus index and spleen index in (**D**) and (**E**). The data of each group were taken for six mice. * *p* < 0.05, ** *p* < 0.01, *vs.* alkaloids or triterpenes alone; ^#^ *p* < 0.05, ^##^ *p* < 0.01, *vs.* blank control group; *^$$^* *p* < 0.01, CTX *vs.* alkaloids, triterpenes or their combination.

The tumor growth inhibition activity of alkaloids and triterpenes and their combined effect *in vivo* were studied by intragastric administration to Lewis cell-bearing mice at a dose of 7.5, 15 and 30 g raw material/kg/d for 14 consecutive days, starting from the day after inoculation with Lewis cells. As depicted in Figure 4A,B, alkaloids, triterpenes and their combination dramatically reduced the tumor growth as compared to the blank control (*p* < 0.05, *p* < 0.01). The rates of inhibition of alkaloids at the dose of 7.5, 15 and 30 g raw material/kg/d were 35.7 ± 5.3%, 46.3 ± 7.3% and 59.5 ± 5.8%, while for triterpenes they were 29.4 ± 5.1%, 41.6 ± 4.1% and 50.8 ± 5.4%. More importantly, the tumor weights in the combination group were lower than those of the alkaloids or triterpenes alone (*p* < 0.05). The rate of inhibition of their combination at the doses of 7.5, 15 and 30 g raw material/kg/d were 50.2 ± 6.9%, 61.5 ± 4.7% and 72.9 ± 6.2%. Additionally, a significant increase in tumor inhibition was observed in their combination group compared to those of alkaloids or triterpenes alone (*p* < 0.05) (Figure 4C). *In vivo* studies clearly showed that alkaloids or triterpenes alone have anti-tumor activity, while their combination was more effective, which indicated the synergistic anti-lung cancer activity of the ALS alkaloids and triterpenes.

### 2.4. The Combination of Alkaloids and Triterpenes Enhances Thymus and Spleen Indexes in Tumor-Bearing C57BL/6 Mice

The thymus index and spleen index were calculated to evaluate the immunomodulatory activity of alkaloids, triterpenes and their combination in tumor-bearing C57BL/6 mice. As shown in Figure 4D,E, the thymus index and spleen indexes of mice in the CTX group were decreased significantly as compared to those of the blank control (*p* < 0.01). Although there is no statistically significant difference between alkaloids, triterpenes, their combination and blank control, the mean thymus indexes and spleen indexes were higher than those of the blank control. Interestingly, the combination of alkaloids and triterpenes significantly enhanced the thymus index and spleen index as compared to alkaloids or triterpenes alone (*p* < 0.05). Furthermore, the oral administration of alkaloids, triterpenes and especially their combination (7.5, 15, 30 g raw material/kg/d), significantly increased the thymus index and spleen index, compared to those of the CTX (20 mg/kg/d) (*p* < 0.05, *p* < 0.01). Our findings demonstrate that alkaloids, triterpenes and especially their combination contribute to the immunostimulation in tumor-bearing C57BL/6 mice.

The immune system plays an important role in the occurrence and development of cancer. In recent years, new therapeutic targets for the immune checkpoint pathways have been found to make immunotherapy a reality in the treatment of lung cancer [14,21]. Tumor cells may escape from host immune system scrutiny and suppress the activation of immune responses. In addition, although treatment methods like surgery, irradiation, and chemotherapy have improved, the serious side effects of these methods limit their use in clinic [22]. In the present experiments, the spleen and thymus indexes were significantly inhibited by the CTX treatment. This could be related to the inhibition effects of CTX on the immune function in tumor-bearing mice [23]. Whether administered alone or combination with ASL leaves alkaloids and triterpenes, spleen and thymus indexes were increased dramatically as compared to the model group. Interestingly, the combination of alkaloids and triterpenes showed the highest immune organ index. Thus, the antitumor activity of the extract could be exerted, at least in part, due to immunostimulation. These compounds may provide significant immunomodulatory activity against lung cancer to maximal clinical benefit.

### 2.5. The Effect of Alkaloids, Triterpenes and Their Combination on TNF-α and IL-6 Level Increases

The potential immunomodulatory activities of alkaloids, triterpenes and their combination were determined by assessing the cytokine-production profiles by ELISA. As shown in Table 2, compared with those of the CTX and blank control groups, the IL-6 and TNF-α levels of mice treated with alkaloids, triterpenes and their combination were significantly higher. This suggested that the alkaloids or triterpenes exerted antitumor immunity activity. Moreover, their combination showed stronger antitumor immunity activity than that exerted by the alkaloids or triterpenes alone.

**Table 2:** Comparison of TNF-α and IL-6 levels in the serum of tumor-bearing mice.

Groups	Dose (raw material/kg/d)	TNF-α (pg/mL)	IL-6 (pg/mL)
Blank	−	46.33 ± 1.98	34.56 ± 3.76
CTX	20 mg/kg/d	40.35 ± 2.36 *^$$^*	28.44 ± 4.59 *^$$^*
Alkaloids	7.5 g	53.73 ± 3.62	42.66 ± 2.84
	15 g	59.57 ± 2.93 ^#^	47.16 ± 2.78 ^#^
	30 g	62.16 ± 2.54 ^##^	51.64 ± 1.77 ^##^
Triterpenes	7.5 g	52.44 ± 2.18	41.75 ± 2.67
	15 g	58.39 ± 1.15 ^#^	48.95 ± 3.00 ^#^
	30 g	62.05 ± 1.69 ^##^	51.41 ± 1.68 ^##^
Alkaloids and triterpenes	7.5 g	57.33 ± 2.30 ^#^	46.97 ± 2.75
	15 g	61.70 ± 1.42 ^*##^	52.69 ± 3.02 ^*##^
	30 g	65.38 ± 1.95 ^**##^	56.83 ± 4.25 ^**##^

^*^ *p* < 0.05, ^**^ *p* < 0.01, *vs.* alkaloids or triterpenes alone; ^#^ *p* < 0.05, ^##^ *p* < 0.01, *vs.* blank control group; *^$$^* *p* < 0.01, *vs.* alkaloids, triterpenes or their combination. 7.5 g, 15 g and 30 g raw material/kg was equivalent to 17.1 mg/mL alkaloids extract or 13.5 mg/mL triterpenes extract or their combination 30.6 mg/mL extract (each mouse for 0.2 mL).

Since the treatment with alkaloids, triterpenes or their combination increased the thymus and spleen indexes, the antitumor activity was thought to be exerted via immunostimulation [24]. Induction of cytokine production (IL-6 and TNF-α) is responsible for enhancing the antitumor immunity. The stimulation of the immune system has been shown to play an important role in the pathogenesis of tumor growth [25]. Alkaloids and triterpenes were able to stimulate the immune response by increasing the expression of IL-6 and TNF-α, and the better antitumor activity of their combination could be related to their up regulation of the expressions of IL-6 and TNF-α.

### 2.6. Annexin-FITC and PI Double Staining for Apoptosis Analysis

**Figure 5:**
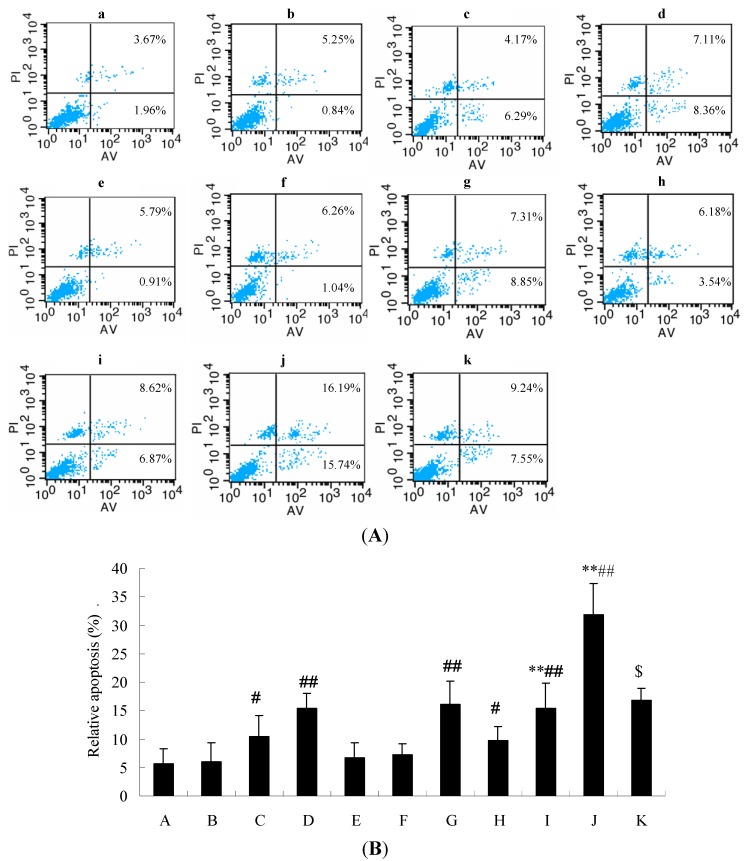
Annexin-V/PI double-staining assay for apoptosis. Cells were treated with (**a**) blank control; (**b**–**d**) alkaloids (1, 2 and 3 mg raw material/mL); (**e**–**g**) triterpenes (1, 2 and 3 mg raw material/mL); (**h**–**j**) their combination (1, 2 and 3 mg raw material/mL); (**k**) positive control cisplatin (10 μg/mL) for 36 h and then co-stained with PI- and FITC-conjugated annexin V. (**A**) Representative apoptotic images; (**B**) Relative apoptosis rates (%). The data are taken for individual experiments (n = 3). * *p* < 0.05, ** *p* < 0.01, the combination of alkaloids or triterpenes (3 mg raw material/mL) *vs.* alkaloids or triterpenes alone (3 mg raw material/mL); the combination of alkaloids or triterpenes (2 mg raw material/mL) *vs.* alkaloids or triterpenes alone (2 mg raw material/mL); ^#^ *p* < 0.05, ^##^ *p* < 0.01, *vs.* blank control group; *^$^* *p* < 0.05, *vs.* alkaloids, triterpenes (1 or 2 mg raw material/mL) or their combination (1 or 3 mg raw material/mL).

Annexin-FITC and PI double staining was performed to evaluate the induction of alkaloids, triterpenes and their combination on A549 cell apoptosis. As shown in Figure 5, after exposure to agents for 36 h, alkaloids, triterpenes or their combination (1, 2 and 3 mg raw material/mL) may significantly increase the apoptosis ratio in a concentration-dependent manner as compared to the blank control. More importantly, the combination of alkaloids and triterpenes has a higher apoptosis ratio than those of the alkaloids or triterpenes alone in A549 cells (*p* < 0.05). For the positive control cisplatin, the apoptosis rate was lower than the combination of these two components at 3 mg raw material/mL. This might be associated with a stronger cytotoxicity. The result demonstrated that alkaloids and triterpenes might display a synergistic action contributing to the induction of apoptosis in A549 cells.

### 2.7. Cell Cycle Analysis

**Figure 6:**
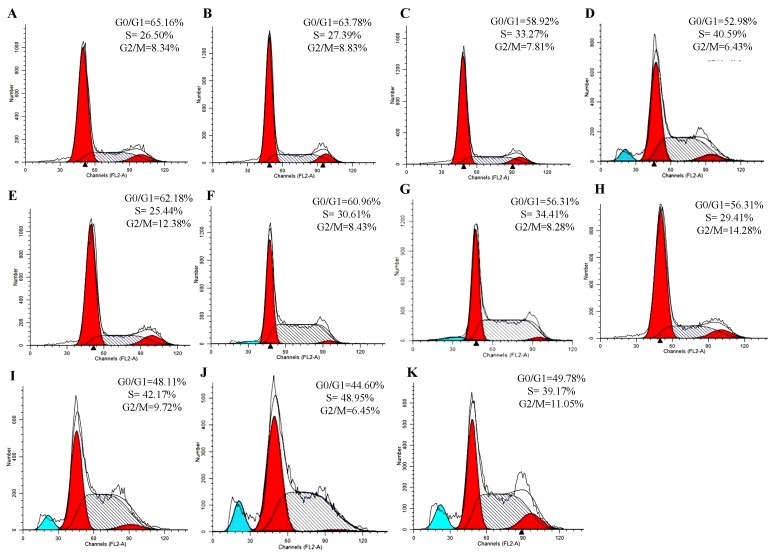
Cell cycle analysis of A549 cells treated with the extract by using flow cytometry. (**A**) blank control; (**B**–**D**) alkaloids (1, 2 and 3 mg raw material/mL); (**E**–**G**) triterpenes (1, 2 and 3 mg raw material/mL); (**H**–**J**) their combination (1, 2 and 3 mg raw material/mL); (**K**) positive control Cisplatin 10 μg/mL. After being treated for 36 h, cells were stainned with PI (10 μg/mL) and RNase (10 μg/mL). The cell-cycle distribution was analyzed by FACS.

To establish whether the ability of alkaloids, triterpenes or their combination to induce apoptosis was related to cell-cycle arrest, the cell cycle distribution of A549 cells was determined by flow cytometry after 36 h treatment with alkaloids, triterpenes or their combination (1, 2 and 3 mg raw material/mL). In the control group, the major cell population was found to be in the G0/G1 phase (65.16%), with a low percentage of cells in the G_2_/M phase (8.43%; Figure 6 and Table 3).

Treatment with alkaloids, triterpenes or their combination (1, 2 and 3 mg raw material/mL) resulted in a significant decrease in the numbers of cells in the G_0_/G_1_ phase and G_2_/M phase whereas an increase in the percentages of cells in the S phase (Figure 6 and Table 3) was seen. Interestingly, a higher decrease in the G_0_/G_1_ phase and an increase in the S phase of cells was found in the combination of alkaloids and triterpenes than with either class alone. This finding indicated that alkaloids, triterpenes or their combination induced apoptosis in A549 cells by causing S cell cycle arrest. Especially, the combination of alkaloids and triterpenes has a significant synergistic effect.

Flow cytometric analysis showed a slight decrease in the G_0_/G_1_ cell population and increase in the S cell population in the alkaloids, triterpenes or their combination-treated groups. The potent anti-lung cancer activity of the combination treatment might be attributed to the synergistic apoptosis mechanism. Generally, the cell cycle arrest is associated closely with apoptosis, that is, the occurrence of cell cycle arrest leads to cell apoptosis, which includes numerous signaling molecules and regulatory proteins [26]. Apoptosis can be initiated via two classic pathways: the death receptor-mediated extrinsic pathway and mitochondria-mediated intrinsic pathway [26]. Therefore, proteins involved in these two pathways were investigated in this study. Our results demonstrated that the induction apoptosis of alkaloids, triterpenes and their combination might be related to the decrease of the anti-apoptotic protein Bcl-2 level and the increase in the cleavage of caspase-8 in A549 cells.

**Table 3:** Regulation of cell cycle.

Groups	Dose (raw material/mL)	G_0_/G_1_ (%)	S (%)	G_2_/M (%)
Blank control	−	65.1 ± 2.6	26.5 ± 4.4	8.3± 1.4
Cisplatin	10 μg/mL	49.8 ± 1.9 ^$$^	39.2 ± 3.8 ^$$^	11.1 ± 2.8^$^
Alkaloids	1 mg	63.8 ± 2.4	27.4 ± 2.9	8.8 ± 1.5
	2 mg	58.9 ± 3.3	33.3 ± 3.6	7.8 ± 1.7
	3 mg	52.2 ± 2.8	41.7 ± 2.5	6.4 ± 2.2
Triterpenes	1 mg	62.2 ± 1.4	25.4 ± 4.6	12.4 ± 1.6
	2 mg	61.0 ± 2.7	30.6 ± 3.9	8.4 ± 1.2
	3 mg	56.3 ± 2.9	34.4 ± 2.1	8.3 ± 0.8
Alkaloids and triterpenes	1 mg	56.6 ± 4.7 ^*#^	29.3 ± 5.2	14.1 ± 3.1
	2 mg	48.1 ± 3.2 ^**##^	42.2 ± 3.7 ^**##^	9.7 ± 1.8
	3 mg	44.6 ± 1.3 ^**##^	48.9 ± 4.3 ^*##^	6. 5 ± 2.4 ^##^

The percentage of each phase distribution was determined and expressed as a percentage of the total cell number. ^*^ *p* < 0.05, ^**^ *p* < 0.01, the combination of alkaloids or triterpenes (3 mg raw material/mL) *vs.* alkaloids or triterpenes alone (3 mg raw material/mL, namely); the combination of alkaloids or triterpenes (2 mg raw material/mL) * vs.* alkaloids or triterpenes alone (2 mg raw material/mL); the combination of alkaloids or triterpenes (1 mg raw material/mL) * vs.* alkaloids or triterpenes alone (1 mg raw material/mL); ^#^ *p* < 0.05, ^##^ *p* < 0.01, *vs.* blank control group; *^$^ p* < 0.05, *vs.* blank control group.

### 2.8. Apoptosis-Related Protein Expressions

**Figure 7:**
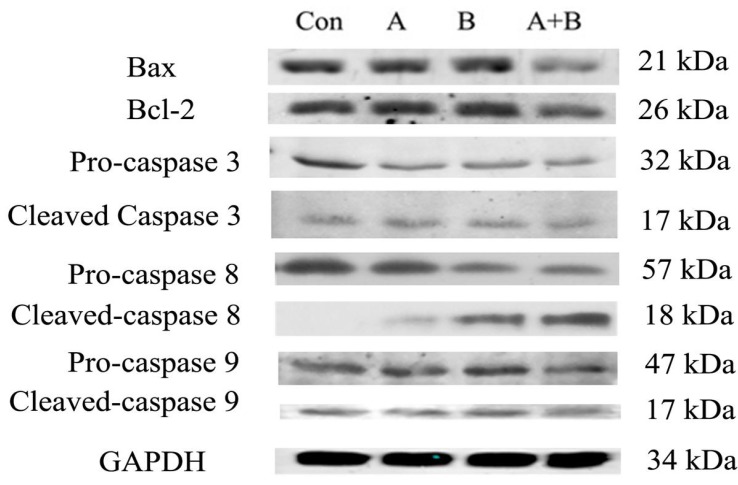
The apoptosis-related proteins level by western blot analysis. After being incubated with alkaloids (**A**), triterpenes (**B**) and their combination (**A** + **B**) (2 mg raw material/mL), cells were incubated with primary antibodies pro-casp3, cleaved-casp3, pro-casp8, cleaved-casp8, pro-casp9, cleaved-casp9, Bcl-2 and Bax (1:1000) and then incubated with secondary antibody IgG-HRP. Immune complexes were detected using an enhanced chemiluminescence system. GAPDH (1:1000) was used as the internal reference.

The possible signaling pathways involved in the induction of alkaloids, triterpenes or their combination on the apoptosis of A549 cancer cells were determined by conducting western blotting analysis of apoptosis-related proteins, including pro-casp3, cleaved-casp3, pro-casp8, cleaved-casp8, pro-casp9, cleaved-casp9, Bcl-2 and Bax. In this experiment, a single dose of alkaloids, triterpenes or their combination (2 mg raw material/mL) was used for western blotting analysis. As depicted in Figure 7, the treatment with alkaloids, triterpenes or their combination led to the cleavage of caspase-8 with the decrease in pro-casp8 level, indicating the activation of the apoptosis pathway. Importantly, the combination treatment with alkaloids and triterpenes was more potent than alkaloids or triterpenes alone in inducing the apoptosis pathway. The expressions of Bax and Bcl-2 were not obviously altered in cells treated by alkaloids or triterpenes alone, however, this alteration was evidently observed in the combination treatment, which might be associated with the action of alkaloids and triterpenes together. For cleaved caspase-3, no more changes were observed which resulted from the balance of the regulation of targets via different pathways. The above findings indicated that alkaloids or triterpenes of ASL leaves might have significant synergistic effect to induce the apoptosis of A549 cells by regulating pro-casp8 and caspase-8 levels.

Lung cancer is the most malignant cancer, with a high morbility and mortality throughout the World [27]. However, current therapeutic approaches for this cancer are still very limited. Although anti-cancer drugs used in clinic are effective, their serious adverse effects limit their use. Medicinal plants and natural products of plant origin are known to have anticancer properties and exhibit low toxicity to normal tissues [28]. Additionally, multiple components of medicinal plants and natural products of plant origin may result in the combinative effect contributing to the anti-cancer activities [29,30,31]. In previous studies, the anti-cancer property of alkaloids and triterpenes of ASL has been confirmed *in vitro*. Our results showed that alkaloids or triterpenes could inhibit the tumor growth in tumor-bearing C57BL/6 mice and the proliferation of A549 cells. More importantly, the combination of alkaloids and triterpenes could inhibit significantly tumor growth *in vivo* and cell proliferation *in vitro*. This result supports the fact that the multiple components might contribute to the enhancement of efficacy by their synergistic effect.

## 3. Experimental

### 3.1. Materials

Dried leaves of *Alstonia scholaris* (Linn.) R. Br. were obtained from Yunnan Plant Technology Co. Ltd. (Yunnan, China), and authenticated as *Alstonia scholaris* (Linn.) R. Br. by Prof. D K Wu from Nanjing University of Chinese Medicine. The voucher specimens (No. ACM20110319) were deposited in Department of Natural Medicinal Chemistry, Jiangsu Provincial Academy of Chinese Medicine. The leaves (5 kg) was extracted by 85% ethanol (100 L) with a reflux extraction system (1.5 h/time, two times). The 85% ethanol ASL extract (2 mg/mL concentration) was loaded on AB-8 type macroporous resin column and eluted with a gradient of 0%, 10%, 20%, 30%, 40%, 50%, 60%, 70%, 80%, and 95% ethanol. The 60% ethanol eluate was the alkaloid fraction, while the 80% ethanol eluate was the triterpenoids fraction. Finally, alkaloids (28.5 g) and triterpenoids (22.5 g) were obtained. The yields of alkaloids and triterpenoids were 0.57% and 0.45%, respectively. Total alkaloids (purity ≥ 77.9%) and total triterpenoids (purity ≥ 74.4%) of ASL were prepared by our laboratory. Antibodies to caspase-3, caspase-8, caspase-9, Bcl-2, Bax and glyceraldehyde-3-phosphate dehydrogenase (GAPDH) were purchased from Cell Signaling Technology (Beverly, MA, USA). Horseradish peroxidase (HRP)-conjugated secondary antibodies were obtained from Santa Cruz Biotechnology (Santa Cruz, CA, USA). Annexin V-FITC/PI apoptosis detection kit was purchased from KeyGEN Biotechnology Co. Ltd. (Nanjing, China). HPLC grade acetonitrile and phosphate were purchased from TEDIA (Fairfield, OH, USA). Cisplatin and cyclophosphamide (CTX) were purchased from Nanjing Pharmaceutical Co., Ltd. (Nanjing, China). Other reagents are analytical grade and from commercial sources.

### 3.2. Cell Culture

The human lung adenocarcinoma cell line A549 and mouse Lewis lung carcinoma cells were obtained from KeyGEN Biotech. A549 cells were maintained in Dulbecco’s modified Eagle’s medium (DMEM) supplemented with 10% fetal calf serum (FCS) (GIBCO), 1% nonessential amino acids, 100 U/mL penicillin, and 100 μg/mL streptomycin. Lewis cells were cultured in Roswell Park Memorial Institute (RPMI)-1640 medium (GIBCO) containing 10% FCS, 100 U/mL penicillin, 100 μg/mL streptomycin. These cells were maintained in a humidified atmosphere of 5% CO_2_ at 37 °C. Medium was changed every two days.

### 3.3. HPLC Analysis

High-performance liquid chromatography (HPLC) was performed using an Agilent 1200 instrument (Agilent Technologies, Palo Alto, CA, USA) equipped with a photodiode array detector (DAD), an autosampler, column compartments and Agilent Chemstation software. The samples were performed on a Zorbax SB-C_18_ column (250 mm × 4.6 mm, 5 μm). For alkaloid analysis, the mobile phase consisted of acetonitrile (A) and 0.5% (v/v) ammonia solution (B); the gradient elution of 20% A was maintained for 0–5 min; 20%–40% A, for 5–20 min; 40% A, for 20–24 min; 40%–60% A, for 24–25 min; and 60% A, for 25–35 min. The detection wavelength was 287 nm. For triterpenes analysis, the mobile phase consisted of acetonitrile (A) and 0.1% phosphate buffer (B, pH = 7.6); the gradient elution of 45% A was maintained for 0–20 min; 45%–80% A for 20–21 min, and 80% A for 21–35 min. The detection wavelength was set at 220 nm. The flow rate of all analysis was kept at 1.0 mL/min. Aliquots of 20 μL were injected.

### 3.4. LC/MS/MS Analysis

The MS analysis was performed on a Finnigan LCQ Fleet ion trap mass spectrometer (Thermo Finnigan, San Jose, CA, USA) equipped with electro spray ionization (ESI) interface. Nitrogen (N_2_) was used as the nebulizing gas and helium (He) as the collision gas. Mass analysis was conducted in positive ion mode. Detailed parameters were set as follows: the flow rate of sheath gas (N_2_) was 40 a.u. while auxiliary gas (N_2_) was 10 a.u.; ion spray voltage was kept at −4.5 kV; capillary temperature was set at 300 °C; capillary voltage and tube lens offset voltage were kept at −22 V and −60 V, respectively. The range of full-scan MS data were recorded within the 100–800 amu range.

### 3.5. MTT Assay for Cell Viability

Cell viability was determined using the 3-(4,5-dimethylthiazol-2-yl)-2,5-diphenyltetrazolium bromide (MTT) assay. Briefly, A549 cells in the logarithmic growth phase were plated at a density of 0.6 × 10^4^ cells/well in 96-well plates. At 24 h of incubation, the cells were treated with alkaloids and triterpenes extract (2.5, 5, 12.5 and 25 μg/mL) for IC_50_ value. Additionally, alkaloids, triterpenes and their combination of 1, 2 and 3 mg raw material/kg, (calculated as raw material) were used for the synergistic action. After incubation for 36 h, MTT (5 mg/mL, 10 μL) was added to each well, and the plates were incubated for 4 h at 37 °C. Subsequently, the supernatant was discarded, and dimethylsulfoxide (DMSO, 100 μL) was added to each well. The absorbance value at 550 nm was measured using a microplate reader (Thermo Labsystems, Helsinki, Finland). Any interference by particle fluorescence was monitored. All experiments were performed thrice.

### 3.6. Lewis Tumor-Bearing C57BL/6 Mice

Male and female C57BL/6 mice (18–22 g) were obtained from the SLAC Lab Animal Center (Shanghai, China). The animal experiment protocol was reviewed and approved by the Institutional Animal Care and Use Committee of the Jiangsu Provincial Academy of Chinese Medicine. Lewis cells were trypsinized and resuspended in PBS (1 × 10^7^ cells/mL). Each mouse was subcutaneously injected 0.2 mL Lewis cells suspension in the right anterior limb as reported previously [32]. The mice were randomly divided into 11 groups: blank control, alkaloids, triterpenes and their combination (7.5, 15 and 30 g raw material/kg/d), and positive control cyclophosphamide (CTX, 20 mg/kg/d). The extracts of alkaloids, triterpenes and their combination which were dissolved in 0.9% NaCl solution were administered orally (the extract was prepared 17.1 mg/mL concentration for alkaloids while 13.5 mg/mL for triterpenes and 30.6 mg/mL for their combination; each mouse was administered orally with 0.2 mL volume. However, the amount of the extracts was calculated as raw material). The blank control group was intragastrically administered the vehicle (0.9% NaCl solution). Positive control (cyclophosphamide) was administered every day by intraperitoneal injection (i.p.). On the 14th day, after being sacrificed, the tumors, spleen and thymus of mice were removed and weighed. The tumor-inhibition rates were calculated using the following formula [33]:

Tumor-inhibitory rate (%) = [(A − B)/A] × 100%

where A and B are the average tumor weights of the control and treated groups, respectively.

Thymus index and spleen index of the mice were calculated to evaluate the immunomodulatory activity in tumor-bearing mice. The immune organic index was calculated as organ weight/body weight [34].

### 3.7. ELISA Assay for IL-6 and TNF-α in Serum

Blood samples were collected and centrifuged at 3500 g for 15 min to obtain the serum. Serum levels of IL-6 and TNF-α were determined using commercially available ELISA kits (KeyGEN Biotech). The detailed procedures were performed according to the manufacturer’s protocols. After reaction, the optical density (OD) of samples was measured by a microplate reader at 450 nm. The contents of IL-6 and TNF-α were calculated by standard curve.

### 3.8. Annexin-V/PI Double-Staining Assay and Cell Cycle

A549 cells (6 × 10^5^/well) were seeded in 6-well plates for conducting the cell cycle distribution or apoptosis analysis. The cells were treated with vehicle (medium) and various concentrations of alkaloids, triterpenes and their combination (1, 2 and 3 mg raw material/kg). After incubation for 36 h, the cells were harvested using trypsin, washed with PBS thrice, and resuspended in 100 μL binding buffer. Annexin V-FITC/PI apoptosis detection kit (KeyGEN) was used for the detection of apoptosis. Annexin-fluorescein isothiocyanate (FITC, 5 µL) and propidium iodide (PI) solution (5 µL) were added, and the cells were incubated in the dark at room temperature for 15 min. Cell apoptosis was analyzed using FACScan flow cytometry (Becton Dickinson, San Jose, CA, USA). Annexin V-positive and PI-negative cells were scored as early apoptotic, and double-stained cells were considered as late apoptotic.

Cell-cycle distribution was determined by staining DNA with PI. Briefly, 6 × 10^5^ cells were washed in PBS and fixed with ice-cold 70% ethanol over night. Cells were incubated with PI (10 μg/mL), with simultaneous RNase (10 μg/mL) treatment, at 37 °C for 30 min. The percentage of cells in each cellular phase was analyzed by FACS.

### 3.9. Western Blot Assay

A549 cells (2 × 10^6^/well) were seeded in 6-well culture dishes overnight and treated with the indicated concentrations of drug (2 mg raw material/mL) for 36 h. The cells were harvested, washed twice with PBS, and lysed for 30 min at 4 °C with ice-cold RIPA buffer (1% NP-40 in 150 mM NaCl, 50 mM Tris, and 2 mM ethylenediaminetetraacetic acid [EDTA]). Equalized amounts of proteins from each sample were subjected to sodium-dodecyl sulfate (SDS)-polyacrylamide gel electrophoresis. Protein bands were then transferred to polyvinylidenedifluoride (PVDF) membranes. The membranes were blocked with 5% (w/v) bovine serum albumin (BSA) for 2 h, washed in TBST thrice, and incubated with primary antibodies pro-casp3, cleaved-casp3, pro-casp8, cleaved-casp8, pro-casp9, cleaved-casp9, Bcl-2 and Bax (1:1000) overnight at 4 °C. The membranes were washed and incubated with the secondary antibody conjugated with IgG-HRP for 1 h at room temperature and then washed in TBST thrice. Immune complexes were detected using an enhanced chemiluminescence system. GAPDH (1:1000) was used as the loading control.

### 3.10. Statistical Analysis

Data are expressed as the means ± standard deviation (SD). One-way analysis of variance (ANOVA) in IBM SPSS 11.5 software (IBM, Armonk, NY, USA) was used for multiple comparisons. The level of significance was set at *p* < 0.05.

## 4. Conclusions

Taken together, the immunomodulatory activity and induction apoptosis of alkaloids and triterpenes extracted from ASL leaves were evaluated for the first time in tumor-bearing C57BL/6 mice and in A549 cells. Importantly, the alkaloids and triterpenes displayed a synergistic effect on tumor growth inhibition *in vivo* and cell proliferation *in vitro* by modulating immunostimulation and inducing apoptosis. The underlying mechanism might be associated with the promotion of cytokine production (IL-6 and TNF-α), induction of cell cycle arrest in the S phase, and progression of apoptotic cell death by down-regulation of Bcl-2 expression and increased caspase-8 cleavage. Our data indicated that the synergistic anti-lung cancer activity of ASL leaves might be beneficial for the prevention and treatment of NSCLC.
